# Inhibition of CD200R1 expression by C/EBP beta in reactive microglial cells

**DOI:** 10.1186/1742-2094-9-165

**Published:** 2012-07-09

**Authors:** Guido Dentesano, Marco Straccia, Aroa Ejarque-Ortiz, Josep M Tusell, Joan Serratosa, Josep Saura, Carme Solà

**Affiliations:** 1 Department of Cerebral Ischemia and Neurodegeneration, Institut d’Investigacions Biomèdiques de Barcelona-Consejo Superior de Investigaciones Científicas (CSIC), Institut d’Investigacions Biomèdiques August Pi i Sunyer (IDIBAPS), C/ Rosselló 161, 6th Floor, Barcelona, E-08036, Spain; 2 Biochemistry and Molecular Biology Unit, School of Medicine, University of Barcelona IDIBAPS, Barcelona, Spain

**Keywords:** Neuroinflammation, Reactive microglia, CD200R1, C/EBPβ, Neuron-microglia communication, *In vitro*

## Abstract

**Background:**

In physiological conditions, it is postulated that neurons control microglial reactivity through a series of inhibitory mechanisms, involving either cell contact-dependent, soluble-factor-dependent or neurotransmitter-associated pathways. In the current study, we focus on CD200R1, a microglial receptor involved in one of these cell contact-dependent mechanisms. CD200R1 activation by its ligand, CD200 (mainly expressed by neurons in the central nervous system),is postulated to inhibit the pro-inflammatory phenotype of microglial cells, while alterations in CD200-CD200R1 signalling potentiate this phenotype. Little is known about the regulation of CD200R1 expression in microglia or possible alterations in the presence of pro-inflammatory stimuli.

**Methods:**

Murine primary microglial cultures, mixed glial cultures from wild-type and CCAAT/enhancer binding protein β (C/EBPβ)-deficient mice, and the BV2 murine cell line overexpressing C/EBPβ were used to study the involvement of C/EBPβ transcription factor in the regulation of CD200R1 expression in response to a proinflammatory stimulus (lipopolysaccharide (LPS)). Binding of C/EBPβ to the CD200R1 promoter was determined by quantitative chromatin immunoprecipitation (qChIP). The involvement of histone deacetylase 1 in the control of CD200R1 expression by C/EBPβ was also determined by co-immunoprecipitation and qChIP.

**Results:**

LPS treatment induced a decrease in CD200R1 mRNA and protein expression in microglial cells, an effect that was not observed in the absence of C/EBPβ. C/EBPβ overexpression in BV2 cells resulted in a decrease in basal CD200R1 mRNA and protein expression. In addition, C/EBPβ binding to the CD200R1 promoter was observed in LPS-treated but not in control glial cells, and also in control BV2 cells overexpressing C/EBPβ. Finally, we observed that histone deacetylase 1 co-immunoprecipitated with C/EBPβ and showed binding to a C/EBPβ consensus sequence of the CD200R1 promoter in LPS-treated glial cells. Moreover, histone deacetylase 1 inhibitors reversed the decrease in CD200R1 expression induced by LPS treatment.

**Conclusions:**

CD200R1 expression decreases in microglial cells in the presence of a pro-inflammatory stimulus, an effect that is regulated, at least in part, by C/EBPβ. Histone deacetylase 1 may mediate C/EBPβ inhibition of CD200R1 expression, through a direct effect on C/EBPβ transcriptional activity and/or on chromatin structure.

## Background

In the presence of neuronal damage, microglial cells acquire reactive phenotypes characterized by both morphological and functional changes [1,2]. Microglial activation is a beneficial response, aimed at re-establishing brain homeostasis and avoiding or minimizing neuronal damage. However, reactive microglial cells produce several factors (pro-inflammatory cytokines, reactive oxygen and nitrogen species) that are typical of an inflammatory response, with potential neurotoxic effects. Consequently, the progression and resolution of microglial activation need to be tightly controlled to avoid the negative secondary effects of excessive or chronic microglial reactivity [3].

In the normal brain, it has been postulated that microglial reactivity is maintained under control by a series of inhibitory mechanisms, in which signals arising from neuronal cells are thought to play an important role (reviewed in [4]). Alterations in these inhibitory signals can result in microglial activation. In the presence of brain tissue injury, microglial cells become activated with a pro-inflammatory phenotype, suggesting that the inhibitory mechanisms have been overcome. In the present work, we focused on the study of one of these inhibitory mechanisms: the CD200-CD200R1 ligand-receptor system.

In the central nervous system (CNS), microglial cells express CD200R1 and CD200 is constitutively expressed mainly by neurons. Results from studies using CD200-deficient mice suggest that this protein plays an important role in the inhibition of the microglial pro-inflammatory phenotype in the normal brain [5-8]. Results from *in vitro* studies also suggest a role for CD200 in the control of microglial activation [9,10]. CD200 expression is decreased in the human brain of patients with multiple sclerosis [11,12], and both CD200 and CD200R1 expression are decreased in the brain of Alzheimer’s disease patients [13]. These observations suggest that the CD200-CD200R1 inhibitory pathway is altered in neurodegenerative disorders affecting the human brain, in which glial activation/neuroinflammation has been suggested to play a role in progression of the neurodegeneration.

Little is known about the molecular mechanisms involved in the regulation of CD200 and CD200R1 expression in physiological and pathological conditions or on the mechanisms involved in the control of the microglial pro-inflammatory response in the presence of CD200R1 stimulation. In terms of CD200, Rosenblum *et al*. [14] described the presence of functional DNA binding sites for tumor suppressor protein p53, a critical regulator of the apoptotic program, in the promoters of the human and mouse CD200 gene and suggested a role for CD200 in the regulation of apoptosis. Gorczynski’s group detected a C/EBPβ binding site in the promoter of the human CD200 gene that is required for the constitutive expression of CD200 [15]. The same group also reported that STAT1α, NF-κB p65 and IRF-1 play a role in the regulation of CD200 inducible expression in human T-cell lines [16]. Lyons *et al*. [9] showed that the anti-inflammatory cytokine IL-4 induced an increase in CD200 expression in rat neurons both *in vivo* and *in vitro*. In contrast, molecular mechanisms controlling the expression of CD200R1 have yet to be identified.

The CCAAT/enhancer binding protein β (C/EBPβ) transcription factor is known to play a role in the control of the expression of genes encoding pro-inflammatory factors in reactive glial cells [17,18]. However, little is known about its role in the regulation of genes encoding anti-inflammatory factors. The objective of the present work was to study whether C/EBPβ plays a role in the regulation of CD200R1 expression in microglial cells. Using glial cultures from wild-type and C/EBPβ-deficient mice and BV2 cells (microglial cell line) overexpressing C/EBPβ, we show that this transcription factor down-regulates the expression of CD200R1 in reactive microglial cells, an effect that is mediated, at least in part, by histone deacetylase (HDAC) 1.

## Methods

### Animals

A colony of C/EBPβ+/- mice [19] on a C57BL/6-129 S6/SvEv background was used to obtain C/EBPβ+/+ and C/EBPβ-/- mixed glial cultures as previously described by Straccia *et al.*[18]. Experiments were carried out in accordance with the Guidelines of the European Union Council (86/609/EU) and following the Spanish regulations (BOE 67/8509-12, 1988) for the use of laboratory animals, and were approved by the Ethics and Scientific Committees of Barcelona University and the Hospital Clínic de Barcelona.

### Cell cultures

Mixed glial cultures were obtained from pools of cerebral cortices of two- to four-day-old C57BL/6 wild-type mice as described by Gresa-Arribas *et al*. [20]. In the experiments with C/EBPβ-deficient mice, C/EBPβ+/+ and C/EBPβ-/- mixed glial cultures were obtained from single 19-day-old embryos from C/EBPβ+/- progenitors as described by Straccia *et al*. [18], due to the infertility of C/EBPβ-/- females and a perinatal death rate of approximately 50% for C/EBPβ-/- neonates. The culture medium consisted of Dulbecco’s modified Eagle medium (DMEM)/F-12 nutrient mixture (Invitrogen, Carlsbad, CA, USA) supplemented with 10% fetal bovine serum (FBS, Invitrogen), 0.1% penicillin-streptomycin (Invitrogen), and 0.5 mg/mL amphotericin B (Fungizone®, Invitrogen). Cells were maintained at 37°C in a 5% CO_2_ humidified atmosphere. Medium was replaced every five to sevendays.

Microglial cultures were prepared from 19 to 21 days *in vitro* (DIV) mixed glial cultures using the mild trypsinization method as previously described by our group [21]. Briefly, the cultures were treated for 30 minutes with 0.06% trypsin in the presence of 0.25 mM ethylenediamine tetraacetic acid (EDTA) and 0.5 mM Ca^2+^. This resulted in the detachment of an intact layer of cells containing virtually all the astrocytes, leaving a population of firmly attached cells identified as >98% microglia. The microglial cultures were used 24 hours after isolation. Flow cytometry studies, qRT-PCR assays, quantitative chromatin immunoprecipitation (qChIP) and co-immunoprecipitation experiments were performed using primary mixed glial cultures due to the limited amount of primary microglial cells usually obtained.

Astroglia-enriched cultures were obtained as described by Saura *et al*. [22].

The mouse microglial cell line BV2 (generated from primary mouse microglia transfected with a v-raf/v-myc oncogene, Blasi *et al*. [23]) was kindly provided by Dr. Elisabetta Blasi (Dip. Scienze Igienistiche, Microbiologiche e Biostatistiche, Modena, Italy). Cells were cultured in Roswell Park Memorial Institute (RPMI)-1640 medium (Invitrogen), supplemented with 0.1% penicillin-streptomycin (Invitrogen) and 10% heat-inactivated FBS. Cells were maintained at 37°C in a 5% CO_2_ humidified atmosphere. Stable clones overexpressing the C/EBPβ LAP isoform were obtained. Briefly, BV2 cells were transfected with 1 μg pCDNA (empty vector) or pCDNA-LAP expression plasmids (Dr. Steven Smale, UCLA, USA) using Lipofectamine 2000 (Invitrogen). Positive clones were selected with Zeocin™ (150 μg/mL) (Invitrogen) associated resistance. Cells were seeded at a density of 5 × 10^4^ cells/mL (1.6 × 10^4^ cells/cm^2^) for immunocytochemistry and at 10^5^ cells/mL (2.4 × 10^6^ cells/cm^2^) for protein and RNA extraction. One day after seeding, the culture medium was replaced with fresh RPMI medium, and the cells were used one day later.

### Treatments

Cells were exposed to 100 ng/mL LPS from *Escherichia coli* (026:B6, Sigma-Aldrich, St. Louis, MO, USA) for different lengths of time.

The HDAC inhibitors suberoylanilide hydroxamic acid (SAHA) and MS-275 (Cayman Chemicals, Ann Arbor, MI, USA) were used at 100 nM, 500 nM, 1 μM and 10 μM. They were added to the cultures one hour before LPS treatment.

### Immunocytochemistry

Cultured cells were fixed with 4% paraformaldehyde in 0.1 M phosphate buffer (pH 7.4) for 20 minutes at room temperature. Non-specific staining was blocked by incubating cells with 10% normal donkey serum (Vector, Peterborough, UK) in PBS containing 1% BSA for 20 minutes at room temperature. Cells were then incubated overnight at 4°C with polyclonal goat anti-CD200R1 (1:50**,** R&D, Abingdon, UK), alone or in combination (mixed glial cultures) with monoclonal rat anti-CD11b (1:200, Serotec, Oxford, UK) or polyclonal rabbit anti-GFAP (1:1000, DAKO, Glostrub, DK) primary antibodies. After rinsing in PBS, cells were incubated for one hour at room temperature with donkey anti-goat ALEXA 488 (1:500) or ALEXA 594 (1:500), alone or in combination with donkey anti-rat ALEXA 594 (1:500) or donkey anti-rabbit ALEXA 546 (1:1000) secondary antibodies (Molecular Probes, Eugene, OR, USA). In the case of mixed glial cultures, cells were permeated with 0.3% Triton X-100 in PBS containing 1% BSA and 10% normal donkey serum for 20 minutes at room temperature following fixation. Cell nuclei were stained with Hoechst 33258 (Sigma). Microscopy images were obtained with an Olympus IX70 microscope (Olympus, Okoya, Japan) and a digital camera (CC-12, Olympus Soft Imaging Solutions GmbH, Hamburg, Germany).

### Isolation of total and nuclear proteins

Total protein extracts were obtained from mixed glial cultures and BV2 cells. One well on a six-well plate was used for each experimental condition. After a cold PBS wash, cells were scraped off and recovered in 100 μL of radioimmunoprecipitation assay (RIPA) buffer per well (1% Igepal CA-630, 5 mg/mL sodium deoxycholate, 1 mg/mL SDS, protease inhibitor cocktail Complete® -Roche Diagnostics, Mannheim, Germany- in PBS). Samples were sonicated, centrifuged for five minutes at 10,400 g and stored at -20°C. The amount of protein was determined using the Lowry assay (Total protein kit micro-Lowry, Sigma-Aldrich).

### Western blot

Total protein extracts (50 μg) or immunoprecipitated samples were denatured (120 mM Tris HCl pH 6.8, 10% glycerol, 3% SDS, 20 mM dithiothreitol (DTT), and 0.4% bromophenol blue, 100°C for five minutes) and subjected to SDS-PAGE on a 10% polyacrylamide minigel, together with a molecular weight marker (Fullrange Rainbow Molecular Weight Marker, GE Healthcare, Little Chalfont, UK), and transferred to a polyvinylidene fluoride (PVDF) membrane (Millipore, Bedford, MA, USA) for 90 minutes at 1 mA/cm^2^. The detection of the proteins of interest was performed as described by Gresa-Arribas *et al*. [20]. Monoclonal mouse anti-C/EBPβ (1:500, Abcam, Cambridge, UK) and monoclonal mouse anti-β-actin (1:50000, Sigma-Aldrich) were used as primary antibodies. Horseradish peroxidase-labelled goat anti-mouse was used as the secondary antibody (1:5000, Santa Cruz Biotechnology, Temecula, CA, USA). The signal was developed with ECL-Plus (GE) and images were obtained using a VersaDoc System (Bio-Rad Laboratories, Hercules, CA, USA). The pixel intensities of the immunoreactive bands were quantified using the % adjusted volume feature of Quantity One 5.4.1 software (Bio-Rad Laboratories). Data are expressed as the ratio between the intensity of the C/EBPβ band and the loading control protein band (β-actin).

### Quantitative real-time PCR

CD200R1 and C/EBPβ mRNA expression was determined in primary glial cultures and BV2 cells six hours after treatments. For isolation of total RNA, one well from six-well culture plates was used per experimental condition, with the exception of primary microglial cultures, where one 75 cm^2^ flask was considered. Total RNA was isolated using a High Pure RNA Isolation Kit (Roche Diagnostics) and 1.5 μg of RNA for each condition was reverse transcribed with random primers using Transcriptor Reverse Transcriptase (Roche Diagnostics). Three nanograms of cDNA were used to perform quantitative real-time PCR. The following primers (Integrated DNA Technology, IDT) were used: 5′-AGGAGGATGAAATGCAGCCTTA-3′ (Forward) and 5′-TGCCTCCACCTTAGTCACAGTATC-3′ (Reverse) to amplify mouse CD200R1 mRNA; 5′-AAGCTGAGCGACGAGTACAAGA-3′ (Forward) and 5′-GTCAGCTCCAGCACCTTGTG-3′ (Reverse) for mouse C/EBPβ. For the normalization of cycle threshold (Ct) values to an endogenous control, the following primers were used: 5′-CAACGAGCGGTTCCGATG-3′ (Forward) and 5′-GCCACAGGATTCCATACCCA-3′ (Reverse) for mouse β-actin mRNA and 5′-GTAACCCGTTGAACCCCATT-3′ (Forward) and CCATCCAATCGGTAGTAGCG (Reverse) for mouse Rn18s mRNA; β-actin and Rn18s mRNA levels were not altered by cell treatment. Real-time PCR was carried out using the IQ SYBR Green SuperMix (Bio-Rad Laboratories) in 15 μL of final volume using an iCycler MyIQ apparatus (Bio-Rad Laboratories). Samples were run for 50 cycles (95°C for 15 seconds, 60°C for 30 seconds, and 72°C for 15 seconds). Relative gene expression values were calculated using the comparative Ct or ΔΔCt method [24] and iQ5 2.0 software (Bio-Rad Laboratories). Ct values were corrected according to the amplification efficiency of the respective primer pair which was estimated from standard curves generated by dilution of a cDNA pool.

### Flow cytometry

CD200R1 protein expression was analyzed in control and LPS-treated (24 hours) mixed glial cultures and BV2 cells. For each experimental condition, 5 × 10^5^ cells were collected and labelled with 10 μg/mL of polyclonal goat anti-CD200R1 (R&D, Abingdon, UK) or goat immunoglobulin G (IgG) (isotype control) in PBS for 20 minutes. Cells were washed once in PBS and incubated with 1 μg/mL of donkey anti-goat ALEXA 488 secondary antibody (Molecular Probes) for 20 minutes. Cell viability was evaluated by incubation with 10 μg/mL propidium iodide (Molecular Probes). CD200R1 expression was analyzed with gating on live cells, using the BD FACSCalibur™ platform (BD, Franklin Lakes, NJ, USA).

### Quantitative chromatin immunoprecipitation (qChIP)

qChIP was performed as described by Straccia *et al*. [18] with some modifications. Briefly, control and LPS-treated (4 hours) primary mixed glial cultures or BV2 cells were cross-linked with formaldehyde (1% vol/vol final concentration) and processed for chromatin extraction. Chromatin shearing resulted in fragments of 500 bp. An aliquot of chromatin sheared from each sample was separated as a loading control for the experiment (input). The rest of the sample was processed for chromatin immunoprecipitation (ChIP) using Dynabeads® protein A (Invitrogen) and 2 μg of polyclonal rabbit anti-C/EBPβ antibody (Santa Cruz Biotechnology) or rabbit IgG (Santa Cruz Biotechnology) as the negative control. DNA was isolated after the elimination of the protein from the immunoprecipitated samples. Input and ChIP samples were analyzed using real-time PCR and SYBR green (Bio-Rad). Three microliters of input DNA (diluted 1/50) and ChIP samples were amplified in triplicate in 96-well plates using the MyIQ Bio-Rad Real Time Detection System, as described in the section on quantitative real-time PCR. The C/EBPβ binding site in the IL-10 promoter was used as a positive control (Liu *et al*., 2003). Match-1.0 (public version, BioBase) and MatInspector (Genomatix) were used to identify C/EBPβ consensus sequences in the 5,000 bp region upstream from the ATG translation start site of the CD200R1 gene. The sequences for each amplified locus and the primers used are shown in Table 1. Samples were run for 45 cycles (95°C for 30 seconds, 62°C for one minute, and 72°C for 30 seconds). For details regarding data analysis see the section on quantitative real-time PCR.

**Table 1:** C/EBPβ putative binding sites in CD200R1 gene promoter and primers used in quantitative ChIP assay

**Box**	**Genomic localization with respect to ATG**		**C/EBPβconsensus sequence**	**Primers**
**1**	−4028	−4014	tgATTTCacaaaat	Fwd: 5′-CATTCCTGCTTCTGTCATGTG-3′
**2**	−3723	−3708	gtctttgGAAATtt	Rev: 5′- GCCCTTACGCTTAACATCCA-3′
**3**	−2793	−2779	tgcttgaGCAATtt	Fwd: 5′-CTTGGGAAAGTTGGGTTGTG-3′
**4**	−2546	−2529	ggcttggGAAAGtt	Rev: 5′-TCCACACCATGGAGTTCATAA-3′
**5**	−1369	−1354	atgtttgGCAAGag	Fwd: 5′-TGAGAGGTGGAGGAGGGTAA-3′
				Rev: 5′-TCCTACCCCTGAGCAAAATG-3′
**6**	−450	−435	tggttagGAAATtt	Fwd: 5′-TCTCACCATGGCATTTTCAA-3′
				Rev: 5′-ATGCCCAAGACAGATGGATG-3′

### Co-immunoprecipitation

Fifty microliters of Dynabeads® protein A (Invitrogen, No.100.01D) were washed three times in PBS with 0.02% Tween-20 and incubated for two hours at 4°C with 4 μg of polyclonal rabbit anti-C/EBPβ (Santa Cruz Biotechnology), or with 4 μg of rabbit IgG (Santa Cruz Biotechnology) as negative control. Two hundred micrograms of protein obtained from chromatin extracts from control and LPS-treated (six hours) mixed glial cultures, as described above in the qChIP protocol, were incubated overnight with the antibody-Dynabeads® complex at 40 rpm on a rotating wheel at 4°C. The immuno complexes were pelleted using a magnetic rack and washed three times with PBS. Beads were removed from the samples by boiling in sample buffer (120 mM Tris HCl pH 6.8, 10% glycerol, 3% SDS, 20 mM DTT, and 0.4% bromophenol blue) for five minutes. Input (1/5 dilution) and immunoprecipitated samples were subjected to SDS-PAGE on a 10% polyacrylamide minigel. Western blot analysis was conducted as described above using monoclonal anti-HDAC1 antibody (1:2500, clone 2E10, Millipore) and monoclonal mouse anti-C/EBPβ antibody (1:500, Abcam). Samples immunoprecipitated with isotype-Dynabeads® or incubated with unlabeled beads were used as negative controls to demonstrate the specificity of the signal obtained.

### Data presentation and statistical analysis

The results are presented as the means + standard error of the mean (SEM). Statistical analyses were performed using one-way or two-way analysis of variance (ANOVA) followed by the Bonferroni post-test when three or more experimental groups were compared. Student’s *t*-test was used to compare two experimental groups. Values of P <0.05 were considered statistically significant.

## Results

### CD200R1 expression in microglial cells decreases in response to the pro-inflammatory stimulus LPS

**Figure 1:**
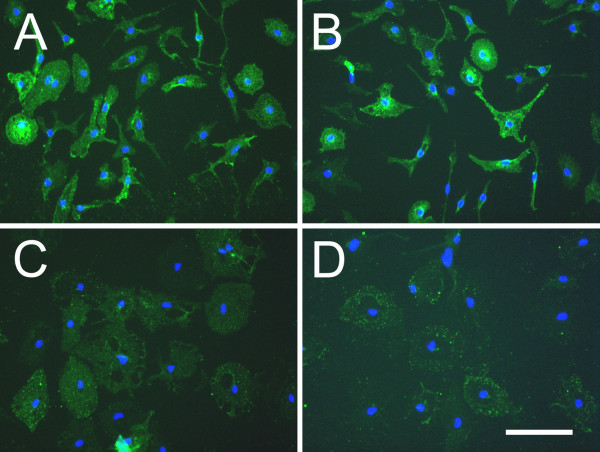
**CD200R1 immunostaining in primary microglial cultures.** (**A**) Control cultures and cultures treated with LPS for 12 hours (**B**), 24 hours (**C**) or 48 hours (**D**). Notice the decrease observed at 24 hours, which was still detected at 48 hours following LPS treatment. Cell nuclei are counterstained with Hoechst 33258. Bar = 100 μm. LPS, lipopolysaccharide.

**Figure 2:**
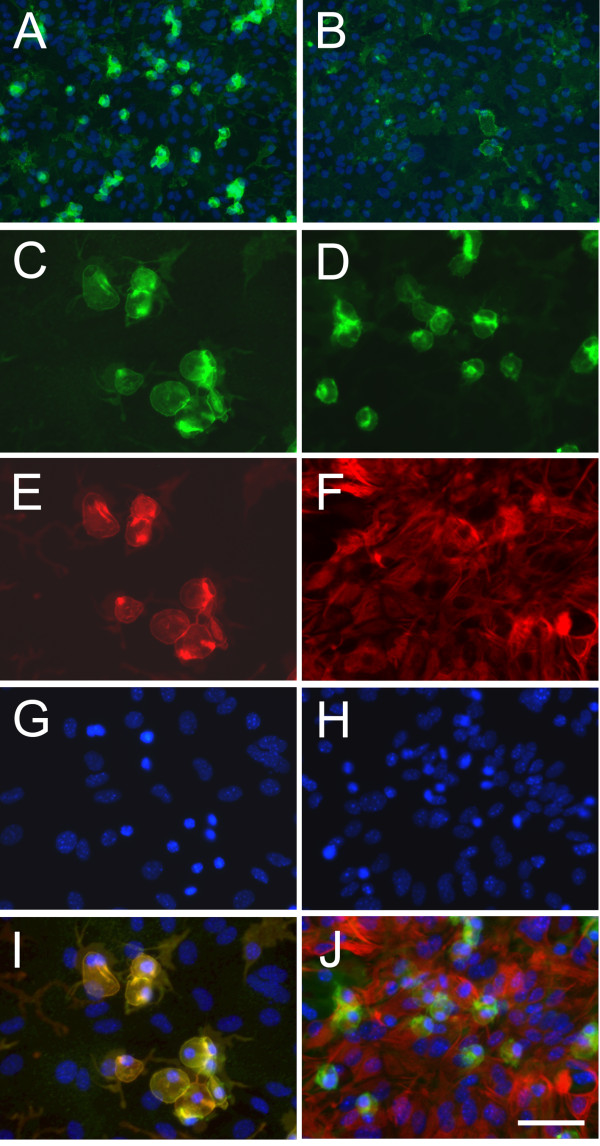
**CD200R1 immunostaining in primary mixed glial cultures.** CD200R1 immunostaining in control primary mixed glial cultures (**A**) and 24 hours after LPS treatment (**B**). The signal was reduced in LPS-treated cultures. CD200R1 immunostaining (**C** and **D**) is localized in anti-CD11b labelled cells (microglia) (**E**) but not in anti-GFAP labelled cells (astrocytes) (**F**). **G** and **H** show Hoechst 33258 staining in the corresponding fields. **I** and **J** show merge images. Bar = 100 μm in A-B and 50 μm in C-J. LPS, lipopolysaccharide.

**Figure 3:**
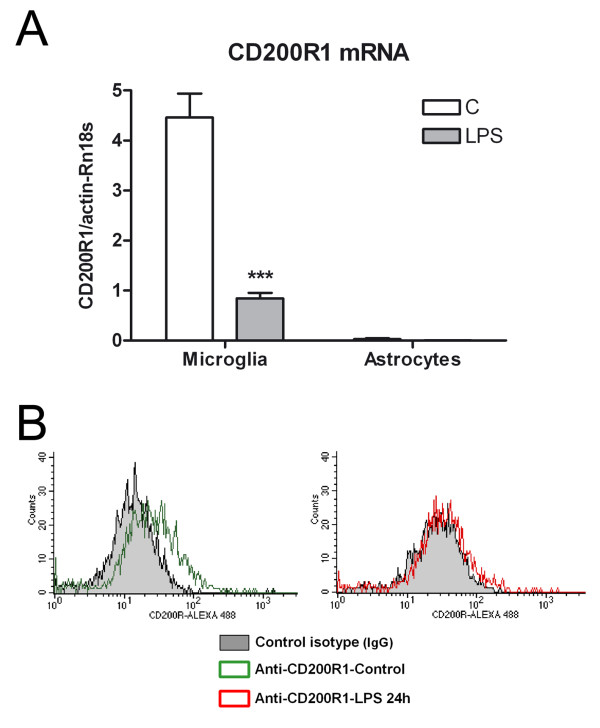
**CD200R1 expression in glial cultures.** (**A**) CD200R1 mRNA was detected in primary microglial cultures but not in astroglia-enriched cultures using qRT-PCR. A clear decrease in CD200R1 mRNA expression was detected in microglial cultures six hours after LPS treatment. Bars are means + SEM of three cultures. ***P <0.001 versus C. Two-way ANOVA and Bonferroni post-test. (**B**) Histogram plots showing CD200R1 staining in mixed glial cultures by flow cytometry. A decrease was observed in cultures treated with LPS for 24 hours (red line) when compared to control cultures (green line). The black line shows control isotype staining. The results are representative of three cultures. ANOVA, analysis of variance; C, control; LPS, lipopolysaccharide; qRT-PCR, quantitative reverse transcriptase polymerase chain reaction; SEM, standard error of the mean.

CD200R1 expression was determined by immunocytochemistry in primary microglial cells in basal conditions and 12, 24 and 48 hours after LPS treatment. CD200R1 immunolabeling was observed in control primary microglial cells, defining the shape of the cells (Figure 1A). No differences in immunolabeling were observed between control and treated microglia 12 hours after LPS (Figure 1B); however, a marked decrease in labeling was observed 24 hours post-treatment (Figure 1C), and was still present at 48 hours (Figure 1D). CD200R1 immunocytochemistry was also evaluated in control and LPS-treated primary mixed glial cultures, composed of astrocytes and microglia. Basal expression of CD200R1 was observed in control cultures (Figure 2A, C and D). CD200R1 immunolabeling in the primary mixed glial cultures was localized in microglial (Figure 2C, E, G and I) but not in astroglial cells (Figure 2D, F, H and J). LPS treatment resulted in a decrease in CD200R1 labeling in the microglial cells (Figure 2B). These results were confirmed at the level of mRNA expression (Figure 3A). Basal expression of CD200R1 mRNA was observed in control primary microglial cultures and a decrease was observed in LPS-treated cultures. No significant CD200R1 mRNA expression was detected in astroglia-enriched cultures. We also detected a decrease in CD200R1 protein expression in LPS-treated primary mixed glial cultures via flow cytometry (Figure 3B).

### The absence of C/EBPβ prevents the inhibition of CD200R1 expression in response to LPS in microglial cells

**Figure 4:**
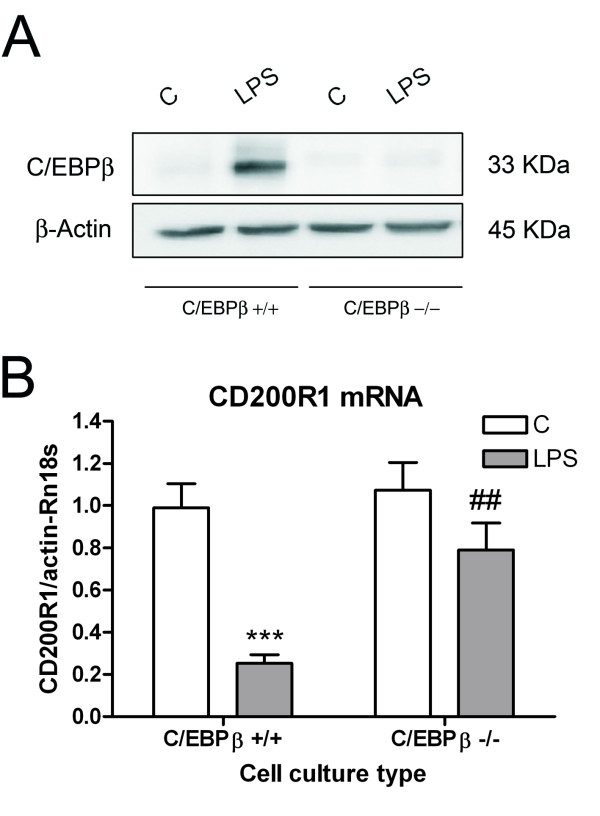
**CD200R1 expression in the absence of C/EBPβ.** (**A**) Western blot showing C/EBPβ protein expression in total protein extracts of primary mixed glial cultures from control and LPS-treated wild-type and C/EBPβ-deficient mice. (**B**) CD200R1 mRNA expression in primary mixed glial cultures from control and LPS-treated (six hours) wild-type and C/EBPβ-deficient mice. The decrease in CD200R1 mRNA expression induced by LPS in wild-type cultures was not observed in C/EBPβ-deficient cultures. Bars are means + SEM of three to four independent experiments. ***P <0.001 versus C; ##*P* <0.01 versus wild type LPS-treated cultures. Two-way ANOVA and Bonferroni post-test. ANOVA, analysis of variance; C, control; C/EBPβ, CCAAT/enhancer binding protein β; LPS, lipopolysaccharide; SEM, standard error of the mean.

To test whether C/EBPβ plays a role in the transcriptional regulation of CD200R1, we analyzed CD200R1 mRNA expression in control and LPS-treated primary mixed glial cultures of wild-type and C/EBPβ-deficient mice using quantitative real-time PCR. We first confirmed that C/EBPβ protein expression was basally detected in wild-type mixed glial cultures and increased after LPS treatment, but was absent in C/EBPβ-deficient cultures (Figure 4A). CD200R1 mRNA expression was significantly lower in wild-type cultures six hours after LPS treatment (Figure 4B). However, this decrease was not observed in LPS-treated C/EBPβ-deficient cultures. This effect occurred without any alteration in CD200R1 mRNA expression in basal conditions in the absence of C/EBPβ.

### C/EBPβ overexpression inhibits CD200R1 expression

**Figure 5:**
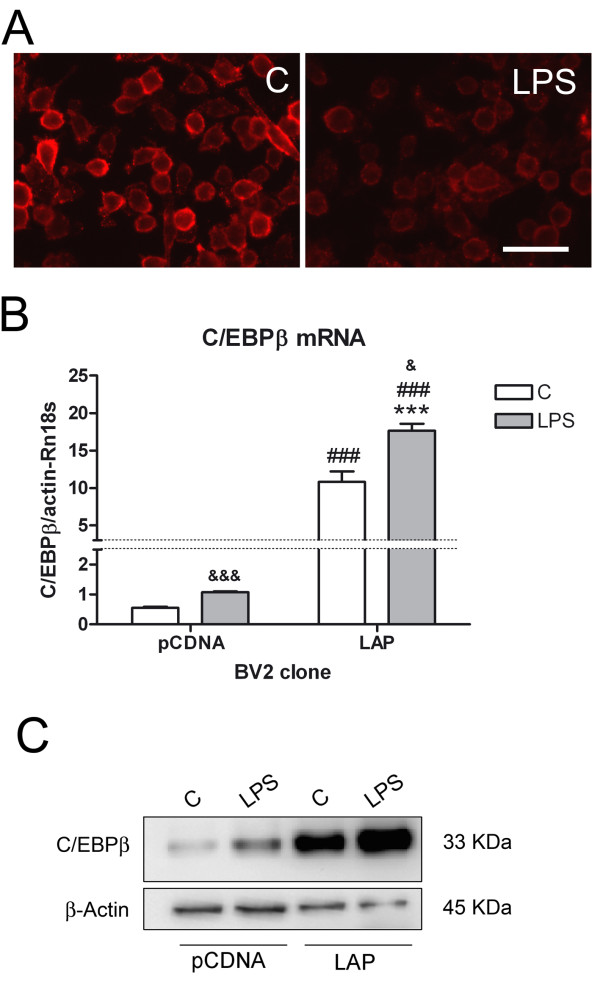
**CD200R1 and C/EBPβ expression in BV2 cells.** (**A**) CD200R1 immunostaining in control and LPS-treated BV2 cells. A clear decrease was observed 24 hours after LPS treatment. Bar = 50 μm. (**B**)C/EBPβ mRNA and (**C)** western blot showing C/EBPβ protein expression in control and LPS-treated (six hours) BV2-pCDNA and BV2-LAP cell clones. Notice the strong increase in C/EBPβ expression in BV2-LAP cells when compared to BV2-pCDNA cells. Bars are means + SEM of three independent experiments. ***P <0.001 versus *C* and ###*P* < 0.001 versus the equivalent experimental condition in BV2-pCDNA cells; two-way ANOVA and Bonferroni post-test. &*P* <0.05 and &&&*P* <0.001 versus C; Student’s *t*-test. ANOVA, analysis of variance; C, control; C/EBPβ, CCAAT/enhancer binding protein β; LPS, lipopolysaccharide; SEM, standard error of the mean.

In order to evaluate whether an increase in the expression of C/EBPβ was sufficient to inhibit CD200R1 gene transcription in microglial cells, we overexpressed C/EBPβ in BV2 cells. We first confirmed the expression of CD200R1 in BV2 cells, and a decrease in this expression after LPS treatment (Figure 5A). We then prepared stable clones of BV2 overexpressing the C/EBPβ LAP isoform (BV2-LAP cells), the most abundant C/EBPβ isoform in glial cells. C/EBPβ overexpression was tested using qRT-PCR and western blot, and significant increases in C/EBPβ mRNA (Figure 5B) and C/EBPβ LAP protein (Figure 5C) were observed in control BV2-LAP cells in comparison to BV2-pCDNA clones (no LAP overexpression). LPS treatment increased C/EBPβ expression in both BV2-pCDNA and BV2-LAP cells.

**Figure 6:**
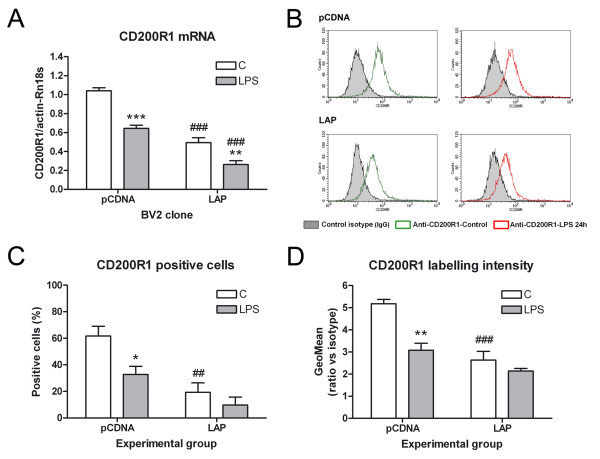
**CD200R1 expression in BV2 cells: effect of C/EBPβ overexpression.** (**A**) CD200R1 mRNA expression in control and LPS-treated (six hours) BV2-pCDNA and BV2-LAP cell clones. (**B-D**) CD200R1 protein in control and LPS-treated (24 hours) BV2-pCDNA and BV2-LAP cells according to flow cytometry. (**B**) Histogram plots, representative of three independent experiments, showing CD200R1 staining in the BV2 cell clones. (**C**) Quantification of CD200R1 positive cells and fluorescence intensity (**D**) in each experimental group. CD200R1 mRNA and protein expression was lower in BV2-LAP cells than in BV2-pCDNA cells, due to an increase in both the number of CD200R1 positive cells and the level of CD200R1 protein expression. Bars are means + SEM of three independent experiments. *P <0.05, **P <0.01 and ***P <0.001 versus C; ##*P* <0.01 and ###*P* <0.001 versus the equivalent experimental condition in BV2-pCDNA cells. Two-way ANOVA and Bonferroni post-test. ANOVA, analysis of variance; C, control; C/EBPβ, CCAAT/enhancer binding protein β; LPS, lipopolysaccharide; SEM, standard error of the mean.

C/EBPβ overexpression resulted in a significant decrease in CD200R1 mRNA in untreated BV2-LAP cells in comparison to untreated BV2-pCDNA cells (Figure 6A). LPS induced a significant decrease in CD200R1 mRNA in both types of clones. A decrease in CD200R1 protein was also observed in BV2-LAP cells versus BV2-pCDNA cells in untreated cultures via flow cytometry (Figure 6B-D). A significant decrease in both the number of cells expressing CD200R1 and the levels of CD200R1 expression was detected in control BV2-LAP cells versus BV2-pCDNA cells.

### C/EBPβ binds to the CD200R1 promoter in response to LPS

**Figure 7:**
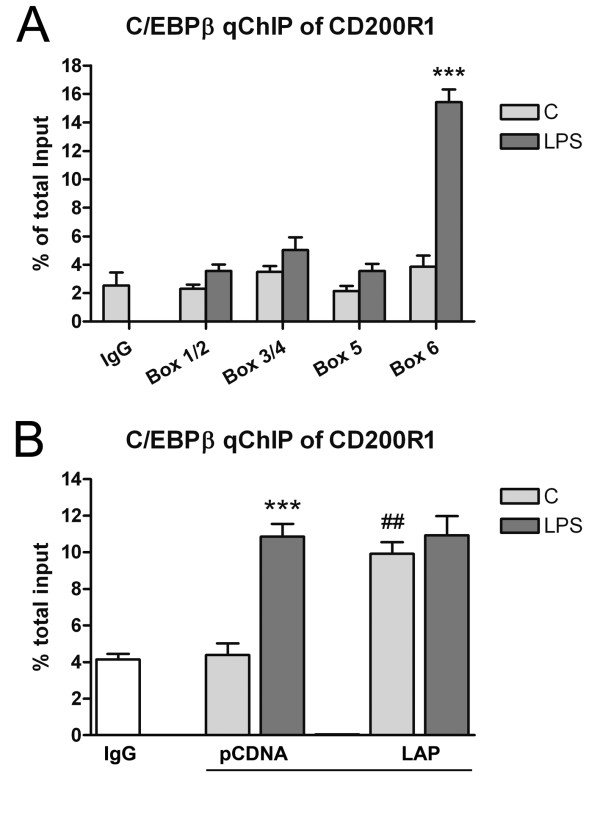
**C/EBPβ binding to CD200R1 gene promoter in glial cells.** (**A**) Analysis of C/EBPβ binding to six putative binding sites of the CD200R1 gene promoter in primary mixed glial cultures by qChIP. Significant binding of C/EBPβ was only observed in box 6 in LPS-treated cultures (four hours). Bars are means + SEM of three independent experiments. ***P <0.001 versus IgG and C. One-way ANOVA and Bonferroni post-test. (**B**) C/EBPβ binding to box 6 of the CD200R1 gene promoter in BV2 pCDNA and BV2-LAP cells. Significant binding of C/EBPβ was observed in LPS-treated (four hours) BV2-pCDNA cells but also in control BV2-LAP cells. LPS treatment did not induce a further increase in C/EBPβ binding in LPS-treated BV2-LAP cells. Bars are means + SEM of three independent experiments. ***P <0.001 versus IgG and C; ##*P* <0.01 versus the equivalent experimental condition in BV2-pCDNA cells. Two-way ANOVA and Bonferroni post-test. ANOVA, analysis of variance; C, control; C/EBPβ, CCAAT/enhancer binding protein β; IgG, immunoglobulin G; LPS, lipopolysaccharide; qChIP, quantitative chromatin immunoprecipitation; SEM, standard error of the mean.

We next studied whether the effect of C/EBPβ on CD200R1 regulation was due to a direct interaction with the gene promoter. We first analyzed the 5,000 bp region upstream from the translation start site of the CD200R1 gene using the bioinformatic softwares Match-1.0 (public version, BioBase) and MatInspector (Genomatix), looking for putative C/EBPβ binding sites. Six putative binding sites (herein referred to as box 1 to 6) were identified, the positions and sequences of which are indicated in Table 1.We then investigated whether C/EBPβ could bind to these sites in a qChIP assay using primary mixed glial cultures. Due to their proximity to each other (less than 500 bp apart), it was impossible to distinguish binding to box 1 and 2 or to box 3 and 4 using our experimental approach. We did not detect any C/EBPβ binding to the CD200R1 gene promoter in untreated primary mixed glial cultures. However, significant binding of C/EBPβ to the CD200R1 gene promoter was observed after LPS-treatment, but only at the binding site closest to the ATG translation start site (box 6) (Figure 7A).

Using our BV2 cell clones, we also assessed whether C/EBPβ overexpression resulted in increased binding of the transcription factor to the CD200R1 gene promoter. As in mixed glial cultures, significant C/EBPβ binding to the CD200R1 gene promoter in BV2-pCDNA cells was only detected after LPS treatment, at the binding site closest to the ATG translation start site (Figure 7B). Nevertheless, significant C/EBPβ binding at the same site was observed in untreated BV2-LAP cells. No differences in C/EBPβ binding were observed between control and LPS-treated BV2-LAP cells.

### C/EBPβ interacts with HDAC1 and determines the inhibition of CD200R1 expression in microglial cells

**Figure 8:**
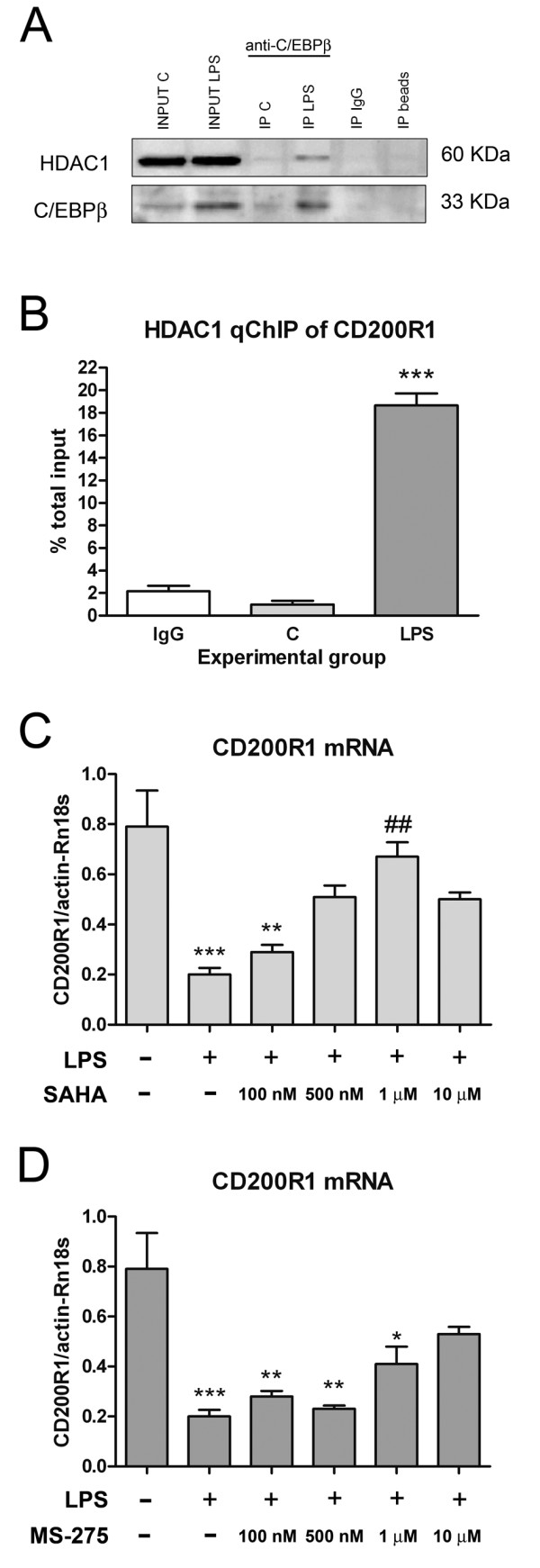
**Involvement of HDAC1 in the inhibition of CD200R1 expression by C/EBPβ.** (**A**) Western blot showing HDAC1 (upper image) and C/EBPβ (lower image) expression in nuclear protein extracts from primary mixed glial cultures before (Input) and after immunoprecipitation (IP) with anti-C/EBPβ antibody. HDAC1-C/EBPβ co-immunoprecipitation was observed in LPS-treated cultures. Samples immunoprecipitated with isotype or unlabeled beads were used as controls to demonstrate the specificity of the signal obtained. (**B**) Analysis of HDAC1 binding to C/EBPβ box 6 of CD200R1 gene promoter by qChIP, showing interaction between HDAC1 and C/EBPβ in LPS-treated cultures. Bars are means + SEM of three independent experiments. ***P <0.001 versus IgG and C. One-way ANOVA and Bonferroni post-test. Effect of the HDAC inhibitors SAHA (**C**) and MS-275 (**D**) on CD200R1 mRNA expression in control and LPS-treated primary mixed glial cultures. Both attenuated LPS-induced inhibition of CD200R1 expression. Bars are means + SEM of three independent experiments. *P <0.05, **P <0.01 and ***P <0.001 versus C; ##*P* <0.01 versus LPS. One-way ANOVA and Bonferroni post-test. ANOVA, analysis of variance; C, control; C/EBPβ, CCAAT/enhancer binding protein β; HDAC1, histone deacetylase 1; IgG, immunoglobulin G; LPS, lipopolysaccharide; qChIP, quantitative chromatin immunoprecipitation; SAHA, suberoylanilide hydroxamic acid; SEM, standard error of the mean.

To further elucidate the mechanism that underlies the C/EBPβ-mediated inhibition of CD200R1 transcription in microglial cells, we analyzed the possible involvement of histone deacetylases. HDAC1 may mediate the effect of C/EBPβ, given the role of this enzyme in histone modifications linked to gene expression inactivation. Using co-immunoprecipitation, we found that HDAC1 interacts with C/EBPβ mixed glial cultures treated with LPS for six hours (Figure 8A). We then analyzed whether HDAC1 was present in box 6 in the CD200R1 promoter using the qChIP assay. We detected binding of HDAC1 in LPS-treated mixed glial cultures (Figure 8B), suggesting that HDAC1 could interact with C/EBPβ to control CD200R1 transcription. Finally, we also investigated whether HDAC1 could play an active role in the transcription of CD200R1, using the HDAC inhibitors SAHA and MS-275. We observed that the inhibition of CD200R1 mRNA expression detected in mixed glial cultures after LPS treatment was partially reverted by both HDAC inhibitors (Figure 8B and C).

## Discussion

The results demonstrate that C/EBPβ is critically involved in the regulation of CD200R1 gene expression in reactive microglial cells. CD200R1 expression decreases in microglia in response to the pro-inflammatory stimulus LPS. However, this effect is not observed in the absence of C/EBPβ and, in contrast, C/EBPβ overexpression results in a decrease in CD200R1 expression in microglial cells in basal conditions. We also show that, in response to LPS, C/EBPβ binds the CD200R1 promoter and that C/EBPβ interacts with HDAC1. These observations suggest that the decrease in CD200R1 expression induced by LPS in microglial cells is due, at least in part, to C/EBPβ transcriptional regulation through a mechanism involving histone deacetylation.

In the CNS, it has been suggested that the CD200-CD200R1 interaction is one of the cell-contact mechanisms involved in the regulation of microglial activity by neurons. Results from studies using CD200-deficient mice and experimental models of inflammatory diseases show that the CD200-CD200R1 interaction keeps microglial cells in a quiescent/surveying phenotype in which the pro-inflammatory response is inhibited, and that microglial cells show increased reactivity when the CD200-CD200R1 signal is impaired [6,8,25-28]. However, the mechanisms of inhibition of pro-inflammatory activity triggered by CD200-CD200R1 signaling in microglial cells have not been characterized. The signal transduction pathways responsible for the inhibitory effects of CD200R1 engagement have only been partially described in mouse mast cells overexpressing CD200R1 differentiated *in vitro*[29] and in the human lymphoma cell line U937 [30]. On the other hand, little is known about the molecular mechanisms regulating CD200 and CD200R1 expression in the CNS. We observed that microglial CD200R1 expression decreases in response to a pro-inflammatory stimulus. This effect would result in decreased interaction between CD200 and CD200R1 in the presence of neurons, which in turn would reduce the inhibitory input microglia receive from neurons in normal conditions. Consequently, one of the mechanisms contributing to the induction of a reactive microglia phenotype by pro-inflammatory factors may be the down-regulation of inhibitory pathways such as CD200-CD200R1 signaling.

Glial activation results in significant changes in the expression of a large number of genes, among them those encoding pro-inflammatory and anti-inflammatory molecules. The expression of these genes must be tightly regulated in order to orchestrate a controlled inflammatory response lasting no longer than necessary. Various transcription factors are involved in the regulation of this expression (NF-κB, AP-1, STATs, PPARs, C/EBPs), resulting in fine regulation of the inflammatory response from start to finish. The transcriptional regulation of CD200R1 has not been studied. We show here that C/EBPβ is one of the transcription factors that is activated by pro-inflammatory stimuli playing a role in the regulation of CD200R1 transcription. C/EBPβ does not appear to play a role in the constitutive expression of CD200R1 in microglial cells, given that we did not detect any alteration in basal levels of CD200R1 mRNA expression in control C/EBPβ-deficient glial cultures. Nevertheless, increased levels of C/EBPβ down-regulate CD200R1 expression, as observed in LPS-treated wild-type glial cultures and not in C/EBPβ-deficient cultures, as well as in BV2 cells overexpressing C/EBPβ. These results suggest that C/EBPβ upregulation in response to LPS contributes to the development of a pro-inflammatory phenotype in microglial cells through the inhibition of CD200R1 transcription.

In recent years, we have been studying the involvement of C/EBPβ in glial activation. Using *in vitro* and *in vivo* experimental approaches we have reported the expression of C/EBPβ in astroglial and microglial cells, and an increase in C/EBPβ expression in reactive glial cells in response to pro-inflammatory stimuli and neuronal death [18,31-33]. This increase was further accentuated in reactive microglial cells of G93A-SOD1 mice (animal model of amyotrophic lateral sclerosis) and also observed in microglial cells in the spinal cord of amyotrophic lateral sclerosis patients [32]. All these results suggest an active role for C/EBPβ in glial activation. C/EBP binding sites have been found in the promoters of many genes encoding pro-inflammatory molecules [34-36]. C/EBPβ regulates the LPS and LPS/IFNγ-induced transcription of IL-6, IL-1β, TNF-α, COX-2 and iNOS genes [18,37-41]. Interestingly, C/EBPβ deficiency has a neuroprotective effect following ischemic [42] and excitotoxic injuries [17], as well as in an *in vitro* model of neuroinflammation [18]. Nevertheless, little is known about the involvement of C/EBPβ in the transcriptional regulation of genes encoding anti-inflammatory molecules, and even less so in CNS cells. Some authors have reported a role for C/EBPβ in induction of the expression of the anti-inflammatory cytokine IL-10 in response to LPS [43,44] or other stimuli [45,46] in macrophages. These observations, together with the results of the present study, suggest that, apart from its role in the expression of pro-inflammatory molecules, C/EBPβ plays a role in the control of the expression of anti-inflammatory molecules, either activating or inhibiting their expression. This constitutes an additional point of regulation of the inflammatory response in glial cells by C/EBPβ.

Several mechanisms may be responsible for the inhibition of CD200R1 gene transcription by C/EBPβ. *In vitro* studies using different cell types have shown that the transactivating activity of C/EBPβ in the transcription of target genes can be inhibited by post-translational modifications such as sumoylation [47,48], phosphorylation [49,50], methylation [51], deacetylation [52,53] and glycosylation [54]. Interestingly, C/EBPβ has been shown to associate with co-repressor complexes containing HDACs in a gene promoter and inhibit gene transcription [55-57]. The acetylation status of histones is a key determinant of transcriptional activity (reviewed in [58]). Histone acetyltransferases and HDACs are the enzymes that reversibly catalyze histone acetylation. Recruitment of histone acetyltransferases to gene promoters is usually associated with the facilitation of gene transcription, while that of HDACs is associated with gene repression. However, it has been shown that a dynamic equilibrium between acetylation and deacetylation is also necessary for transcriptional activity. Using glial cultures, we found that C/EBPβ interacts with HDAC1 and that HDAC1 binds the CD200R1 promoter at a C/EBPβ consensus sequence following LPS treatment. These results, together with the observation that HDAC inhibitors reverse the LPS-induced reduction in CD200R1 expression, suggest that HDAC1 plays a role in the C/EBPβ-mediated repression of CD200R1 transcription observed in microglial cells in response to LPS treatment. However, the possible involvement of other HDACs cannot be ruled out. This mechanism of transcriptional repression could involve both histone deacetylation [55-57], resulting in changes in chromatin structure and consequently in transcriptional activity, and C/EBPβ deacetylation [52,53], resulting in direct changes in transcriptional activity.

In recent years, several *in vitro* studies have shown that HDAC inhibitors down-regulate pro-inflammatory gene expression in macrophages and glial cells [59], as well as in other cell types [60-62], in response to inflammatory stimuli. HDAC inhibitors also attenuate the pro-inflammatory response in experimental models of cerebral ischemia [63] and endotoxemia *in vivo*[61]. Our results suggest that the anti-inflammatory effects of HDAC inhibitors can be mediated, at least in part, by potentiating the transcription of genes involved in keeping the pro-inflammatory response under control, such as CD200R1.

## Conclusions

We describe, for the first time, a molecular mechanism involved in the regulation of the expression of CD200R1 in microglial cells, in which the transcription factor C/EBPβ plays a role. CD200R1 expression decreases in microglial cells in response to a pro-inflammatory stimulus, reducing the input that inhibits the microglial pro-inflammatory phenotype in physiological conditions. We show that C/EBPβ, in addition to its known role in the regulation of the expression of pro-inflammatory genes, can also negatively regulate the expression of genes involved in the inhibition of the pro-inflammatory response, such as CD200R1, thus contributing to the development of the pro-inflammatory phenotype in microglial cells. HDAC1 may mediate the inhibition of CD200R1 expression by C/EBPβ through changes in chromatin structure and transcriptional activity.

## Abbreviations

ANOVA, analysis of variance; bp, base pair; BSA, bovine serum albumin; C/EBPβ, CCAAT/enhancer binding protein β; CNS, central nervous system; Ct, cycle threshold; DIV, days in vitro; DMEM, Dulbecco’s modified Eagle’s medium; DTT, dithiothreitol; EAE, experimental autoimmune encephalomyelitis; FBS, fetal bovine serum; HDAC, histone deacetylase; IFNγ, interferon γ; IgG, immunoglobulin G; IL, interleukin; LPS, lipopolysaccharide; PBS, phosphate buffered saline; PCR, polymerase chain reaction; PVDF, polyvinylidene fluoride; qChIP, quantitative chromatin immunoprecipitation; qRT-PCR, quantitative reverse transcriptase-polymerase chain reaction; RIPA, radioimmunoprecipitation assay; SAHA, suberoylanilide hydroxamic acid; SEM, standard error of the mean; TBS, tris-buffered saline; TNF, tumor necrosis factor.

## Competing interests

The authors declare that they have no competing interests.

## Authors’ contributions

GD performed most of the experiments, analyzed the data and helped write the manuscript. MS obtained and treated the mixed glial cultures in the experiments with C/EBPβ-deficient mice. AEO obtained the pCDNA-BV2 and LAP-BV2 cell clones. JMT and JSe participated in immunocytochemistry experiments. JSa provided critical guidance and contributed to the final version of the manuscript. CS conceived and coordinated the study, provided guidance in the production of data and drafted the manuscript. All authors critically revised and approved the final manuscript.
